# Vaginal and oral use of probiotics as adjunctive therapy to fluconazole in patients with vulvovaginal candidiasis: A clinical trial on Iranian women

**DOI:** 10.18502/cmm.7.3.7803

**Published:** 2021-09

**Authors:** Zahra Vahedpoor, Mahdi Abastabar, Mojtaba Sehat, Parisa Talebian, Tayebeh Felfelian Fini, Zahra Dastanpour, Iman Haghani, Ramtin Chelongarian, Mehdi Nazeri

**Affiliations:** 1 Autoimmune Diseases Research Center, Kashan University of Medical Sciences, Kashan, Iran; 2 Invasive Fungi Research Center, Communicable Diseases Institute, Mazandaran University of Medical Sciences, Sari, Iran; 3 Department of Medical Mycology, School of Medicine, Mazandaran University of Medical Sciences, Sari, Iran; 4 Department of Epidemiology, Faculty of Medicine, Kashan University of Medical Sciences, Kashan, Iran; 5 Obstetrics and Gynecologist, Kashan, Iran; 6 Beheshti Hospital, Kashan University of Medical Sciences, Kashan, Iran; 7 Department of Medical Parasitology and Mycology, Faculty of Medicine, Kashan University of Medical Sciences, Kashan, Iran

**Keywords:** Fluconazole, Probiotic, Vulvovaginal candidiasis

## Abstract

**Background and Purpose:**

Vulvovaginal candidiasis is considered the second most prevalent gynecologic infection among women and one of the main reasons for referring to a gynecologist. During recent decades, probiotic usage has been defined as one of the therapeutic regimens for vaginal candidiasis management, but these findings were controversial. The current study was conducted to determine the effect of fluconazole plus vaginal and oral probiotics supplementation on clinical and mycological improvement of vaginal candidiasis concomitant with antifungal susceptibility of *Candida* species to fluconazole.

**Materials and Methods:**

This double-blind, randomized, placebo-controlled trial was conducted on 76 women with vaginal candidiasis admitted to Naghavi and Imam Reza Gynecology Clinics in Kashan, Central Iran, from July 2017 to March 2020. Patients were diagnosed according to vaginal candidiasis symptoms and positive culture for *Candida* species. The patients were divided into two groups; one of them received fluconazole plus vaginal and oral probiotics, while the other one received fluconazole with placebo. The clinical and mycological findings were recorded before and after the treatment. In vitro, the fluconazole susceptibility test was determined by the microdilution method according to the Clinical and Laboratory Standards Institute (M27-A3) for the baseline *Candida* isolates.

**Conclusion:**

Based on the findings, 35 days after the intervention, a significant reduction was reported in vaginal candidiasis symptoms in the probiotics supplementation group. Although probiotics supplementation therapy was a better mycological cure, compared to the fluconazole with the placebo group, this difference was not significant (68.4% vs. 46.9%, *P*=0.184). Exclusion of resistant and susceptible dose-dependent strain in the regression model demonstrated a significant reduction in positive culture probiotics in the supplementation group.

**Results:**

Oral and vaginal supplementation with probiotics for 4 weeks played a significant role in the elimination of vaginal candidiasis symptoms. Adjustment of clinical and mycological responses with drug resistance patterns of patients could open a promising horizon for probiotics consumption as a complementary treatment.

## Introduction

ungi are the main ingredients of the human microbiome [ 1 ] and are related to approximately 1.7 billion superficial fungal infections (SFIs) and 1.5 million mortality due to invasive fungal infections [ 2 ]. While invasive fungal infections have been the focus of medical mycologists [ 2 ], SFIs are sometimes neglected and considered as mild and easily treatable cases. Vulvovaginal candidiasis (VVC) is one of the most prevalent manifestations of SFIs [ 2 , 3 ], and it is estimated that nearly 75% of women have a history of vaginal candidiasis during childbearing [ 4 ]. More importantly, near to 5% of women have recurrent vulvovaginal candidiasis (RVVC) which is defined as at least four episodes of infection during 1 year, despite antifungal therapy [ 5 ].

It has been estimated that approximately 138 million women suffer from RVVC annually, and this number is predicted to increase to 158 million by 2030 [ 3 ]. This disease can severely affect the quality of life of patients and impose a significant financial burden on them, which exceeds 14.39 billion USD in developed countries [ 3 ]. Since the treatment is costly and time-consuming, in poor communities, where access to physicians and insurance coverage is more limited, the cases are treated by over-the-counter and traditional products. 

Overuse of antibiotics, pregnancy, cystic fibrosis, and diabetes are among the predisposing factors for RVVC and VVC [ 3 ]. In numerous studies, *Candida* albicans are indicated as the dominant species (70-90%), while a minority of infections are attributed to non-albicans *Candida* species, such as *C. glabrata*, *C. tropicalis*, *C. parapsilosis*, *C. kefyr*, *C. guilliermondii*, and *C. krusei* which are naturally more resistant to azoles [ 6 , 7 ]. Symptoms of VVC include physical complaints, such as vaginal discharge, burning urination, vulvar pruritus, dyspareunia, irritation, and psychological problems that harm sexual desire which could lead to the destruction of marital life [ 8 , 9 ].

Despite different predisposing factors for VVC, its pathogenesis has been controversial up to now. Vaginal microbial flora plays an important role in improving vaginal health and preventing vaginal infections [ 10 , 11 ]. Therefore, any disturbance among vaginal microbiota may play a remarkable role in facilitating *Candida* species growth [ 12 , 13 ]. The VVC is treated by numerous intravaginal and oral antimycotic agents, but the cure rates are different according to vaginal candidiasis forms (complicated or uncomplicated) that naturally have different predisposing and causative agents [ 14 ]. Overall, the success rate for the treatment of acute vaginal candidiasis is approximately 80% and different antifungal agents are equally effective [ 5 ]. 

Fluconazole (Diflucan), as a triazole, is one of the first-line treatments for VVC that act through inhibition of ergosterol synthesis, alteration of cellular membranes, and increase of membrane permeability in *Candida* species [ 15 , 16 ]. Fluconazole in both oral and topical form has demonstrated a good efficacy and safety profile for the treatment of candidiasis [ 16 ]. *C. albicans* was reported to be the most common agent of VVC [ 6 , 17 ]. However, in recent decades, the pattern of *Candida* species has changed, accordingly, there has been an increase in the role of non-*C. albicans* *Candida* species as an etiological agent of VVC. This change has been influenced by the widespread use of broad-spectrum antibiotics and the over-the-counter use of antimycotic agents [ 18 ].

The majority of non-*C. albicans* species have an intrinsic resistance or low susceptibility to some antifungal agents; therefore, failure of treatment in fungal infection due to non-*C. albicans* species is not far from expectation [ 19 - 22 ]. According to the theory that any disruption in vaginal microbiota could lead to VVC, different studies were carried out to define a therapeutic and protective role for probiotic lactobacillus [ 9 , 13 , 23 - 25 ]. The ability of probiotics in maintaining and recovering the normal vaginal microbiota, and their potential ability to resist *Candida* gave rise to the concept of using probiotics for the treatment of VVC [ 26 ]. 

During the recent decades, probiotics, known as a part of normal human bacterial flora, have been defined as a new strategy for challenging vaginal candidiasis by different routes, including the reduction of intravaginal pH [ 27 ], improvement of mucosal defense barrier against yeast cells, improvement of vaginal normal flora, and production of specific molecules, such as bacteriocins, extracellular proteins or hydrogen peroxide [ 28 , 29 ]. However, the role of probiotics in the treatment of vaginal candidiasis has been controversial up to now. Results of different studies have indicated the beneficial role of probiotics in the treatment of VVC [ 30 , 31 ]. 

Numerous studies have been performed to determine the therapeutic role of probiotics; nevertheless, most of them had some limitations which could have influenced their outcomes. These limitations included poor design, unverified VVC, and randomization regardless of uncomplicated and complicated VVC caused by non-albicans *Candida* species, particularly *C. glabrata* which are intrinsically resistant to fluconazole. Moreover, some studies did not mention how the episodes of recurrent VVC were identified before their inclusion in the trials. Perhaps they were only diagnosed just through self-diagnosis and vaginal fluid cultures were not obtained [ 13 , 23 , 32 ].

This study was conducted to compare the clinical and microbiological efficacy of treatment with fluconazole and treatment with fluconazole plus oral and vaginal probiotics capsules. Moreover, it aimed to identify the *Candida* species and their susceptibility to fluconazole for further justification of the efficacy of probiotics in the treatment of vaginal candidiasis.

## Materials and Methods

### Trial design and participant 

This study was a randomized, double-blind, placebo-controlled clinical trial registered in the Iranian registry of the clinical trial (http://www.irct.ir: IRCT2016090529710N1) which was conducted at Naghavi and Imam Reza gynecology clinics affiliated to Kashan University of Medical Sciences (KAUMS), Kashan, Central Iran, from July 2017 to March 2020.

The inclusion criteria were VVC based on 2015 Sexually transmitted Diseases Treatment Guidelines and age range of 18-48 years old [ 14 ]. Exclusion criteria were consumption of antifungal drugs (systemic or intravaginal) within 4 weeks before the intervention, diabetes, allergic reactions to fluconazole and probiotics to both administration routes (vaginal or oral), pregnancy, affliction by other vaginal infections, and usage of antibiotics during the intervention.

### Study design

All women who were admitted to Naghavi and Imam Reza Gynecology clinics with signs and symptoms of VVC, such as vulvar burning, vulvar itching, abnormal vaginal discharge, and dyspareunia visited by a gynecologist as well as the patients with approved VVC based on laboratory exams were included in this study. The subjects were randomly divided into two groups. One of the groups received fluconazole plus 150-mg vaginal probiotic (Lactovag) supplements, including Lactobacillus acidophilus 1×109, Lactobacillus plantarum 1×109, Lactobacillus rhamnosus 1×109 Lactobacillus gasseri 1×109 colony-forming unit/day for 14 nights and oral probiotic (Lactofem) supplements, including Lactobacillus acidophilus 1×109, Lactobacillus plantarum 1×109, Lactobacillus fermentum 1×109, Lactobacillus gasseri 1×109 colony-forming unit/day for 30 days (n=38). The other one received fluconazole150-mg plus vaginal and oral placebo for 14 nights and 30 days (n=38).

Placebos and probiotic capsules were identical as they had similar colors, shapes, sizes, and packages made by Zist-Takhmir Pharmaceutical Company (Tehran, Iran). Patients with recurrent VVC took a 150-mg oral dose of fluconazole every 72 h (three doses in total). All patients were recruited after 30-35 days and were reassessed (clinical and mycological) if their treatment had been completed. Randomization was managed using computer-generated random numbers and randomized allocation was conducted by a trained midwife at a gynecology clinic.

### Clinical assessment

All women with one or several signs and symptoms, such as abnormal vaginal discharge, vaginal itching, vaginal burning, and dyspareunia which are considered major complaints of vaginitis, and those who were admitted to the Naghavi and Imam Reza gynecology clinics were visited by a gynecologist. All suspected patients that met the inclusion criteria were introduced for registration of demographic and medical information and vaginal sampling. A vaginal sample was taken by two sterile swabs from vaginal secretions for mycological and vaginal pH assessment.

### Sample size determination

The sample size for this trial was calculated based on a prior study (9). The probiotic-treated group showed significantly less vaginal discharge associated with any of the above-mentioned symptoms of candidiasis and lower positive culture, compared to the placebo group (10.3% vs. 35.6%; *P*=0.03) (10.3% vs. 38.5%; *P*=0.014). The sample size was calculated for both symptoms of candidiasis and positive culture but a greater sample size was considered. The type one (α) and type two errors (β) were determined to be 0.05 and 0.20, respectively. Moreover, 10% for loss to follow-up was added; hence, finally, the sample size was determined 40 subjects per group.

$n=\frac{{\left(z_{\mathrm{1-\alpha /2}}+z_{\mathrm{1-\beta}}\right)}^{2}(p_{1}(1-p_{1})+(p_{2}(1-p_{2}))}{\left(p_{1}-p_{2}\right)}$

### Randomization

Randomization was performed by a simple method and using random numbers generated by computer software (Stat Trek software). In this method, the computer selects random numbers, and randomization and allocation were concealed from the investigators and participants until the final analyses were completed. Randomized allocation was conducted by a trained midwife at a gynecology clinic.

### Mycological assessment

Vaginal pH was assessed using an impregnated swab obtained from the lateral vaginal wall by a pH indicator strip (Macherey-Nagel, Duren, Germany). Further detections were conducted by direct examination (Gram staining and wet mount preparation) and culture. Bacterial vaginitis was ruled out by Amsel criteria, namely clue cells in the Gram staining smear, pH ≥ 4.5, and positive whiff test. In addition, wet mount preparation ruled out trichomoniasis. The culture was performed by inoculation of the impregnated vaginal swab with vaginal discharge on Sabouraud dextrose agar (Biolife, Italy) supplemented with chloramphenicol (0.5μg/ml) based on a previous study [ 33 ]. *Candida* species were identified using morphological and physiological properties and produced color in CHROM agar *Candida* (bioMérieux, Marcy l’Etoile, France). Subsequently, genomic DNA was extracted from all the test isolates and all the strains were identified by the previously-described polymerase chain reaction-restriction fragment length polymorphism method [ 34 , 35 ].

### Antifungal assessment

The antifungal susceptibility testing of fluconazole was assessed by the microdilution method recommended by the Clinical and Laboratory Standards Institute (CLSI, documentM27-A3) [ 36 ].

### Outcomes

Clinical symptoms, including vaginal discharge, vaginal burning, vaginal itching, and dyspareunia, were considered the primary outcomes, and fungal culture was considered the secondary outcomes.

### Ethical statement

The study protocol was approved by Research Ethics Committee, KAUMS, Iran. Written, informed consent for vaginal sampling and subsequent treatments were taken from all participants before enrolment in the study, following the principles of the Helsinki Declaration.

## Results

In total, 70 patients (probiotic [n=32] and placebo [n=38]) completed the trial. In this study, the compliance was noticeable, although 10 patients (intervention: 8, control: 2) did not complete the trial. No adverse effects were observed in patients with VVC following probiotic supplementation. There were no significant differences between the two groups in terms of social demographic information, sexual activity, and type of contraception methods (Opcicon One-Step, intrauterine device, male condom, and natural methods) at the baseline of the trial (Table 1). 

**Table 1:** Baseline demographic characteristics of study groups

Variables	Group		P-value
	Fluconazole and probiotic (n=38)	Fluconazole and placebo (n=32)	
Age (year)	34.61±8.19	33±7.13	0.389
Relapse (year)	3.42±1.88	3.44±2.09	0.928
Sexual activity (week)	1.84±0.86	2.31± 1.35	0.214
Occupation			
Employed (%)	2 (5.3)	4 (11.5)	0.402
Unemployed (%)	36 (94.7)	28 (87.5)	
Contraceptive method			
OCP (%)	3 (7.9)	0 (0)	0.280
IUD (%)	15 (39.5)	20 (62.5)	
TL (%)	2 (5.3)	0 (0)	
Male Condom (%)	12 (31.6)	7 (21.9)	
Natural Methods (%)	4 (10.5)	2 (6.3)	
Not at risk (%)	1 (2.6)	2 (6.3)	
Antifungal susceptibility: ** susceptible≤8µg/ml Dose-dependent susceptible 16-32 8µg/ml Resistance≥64**			
Susceptible (%)	30 (78.9)	29 (90.6)	0.181

Vaginal symptoms, such as abnormal vaginal discharge, vaginal itching, vaginal burning, dyspareunia, and pH were not statistically different between the two groups at the baseline of the study. Therefore, the signs and complaints between the two groups were similar. Rates of infection with vaginal candidiasis due to non-albicans *Candida* species among the probiotic supplementation and the placebo group were 21.1% and 21.8%, respectively, which had no significant difference at the baseline of the study. Moreover, the rate of resistance and dose-dependent susceptible strains between the two groups had no significant difference (*P*=0.181) (Table 1).

It should be mentioned that 35 days after the intervention, vaginal complaints and negative culture (clinical and mycological improvement) increased in both groups. Furthermore, there was a significant difference between the groups before and after the treatment. Moreover, only the patients in the intervention group had a significant difference (*P*=0.021) in terms of the decrease in pH (<4.5) (Table 2).

**Table 2:** Clinical and mycological outcomes of study groups seven days after treatment.

Complaints	Groups								(P-value**)
	Fluconazole and probiotic (n=38)				Fluconazole and placebo (n=32)				OR
	Before treatment		After treatment		Before treatment		After treatment		
	No	%	No	%	No	%	No	%	
Vulvar burning	28	73.7	3	7.9	25	78.1	11	34.4	(0.011)
	*P*<0.001*				*P*=0.001*				6.21
Vaginal discharge	36	94.7	7	18.4	31	96.1	20	62.5	(0.000)
	*P*<0.001*				*P*=0.002*				7.38
Vulvar itching	31	81.6	2	5.3	28	87.5	14	43.8	(0.001)
	*P*<0.001*				*P*<0.001*				13.82
Dyspareunia	19	50	4	10.5	18	56.5	6	18.8	(0.394)
	*P*<0.001*				*P*=0.001*				1.86
pH<4.5	10	26.3	20	52.8	12	40	14	45.2	(0.527)
	*P*=0.021*				*P*=0.815*				1.36
Culture	38	100	13	31.6	32	100	16	53.1	(0.184)
	*P*<0.001*				*P*<0.001*				1.92
*C. albicans*	30	78.9	6	46.2	25	78.1	9	56.2	(0.181)
*C. non- albicans*	8	21.1	7	53.8	7	21.8	7	43.8	3.658
	*P*=0.062*				*P*= 1.00*				

* McNemar's test ; ** Logistic regression

The logistic regression test indicated a significant difference (*P*≤0.05) in burning, discharge, and itching in the probiotic supplementation group, and the odds ratios for these complaints were 6.21, 7.38, and 13.82, respectively. However, dyspareunia and pH < 4.5 were not significantly different between these two groups (*P*>0.05) (Table 2).

Mycological cure (negative culture) between the two groups did not have a significant difference (*P*=0.184). However, the mycological cure (68.4%) in the probiotic supplementation group was higher than in the placebo group. The difference between the ratios of albicans/non-albicans and VVC/RVVC in the two groups was not significant in terms of mycological response, which has not been evaluated in other studies (Table 2).

The minimum inhibitory concentration50 (MIC50) of fluconazole for *Candida* isolates of the two groups was similar (1 µg/ml). The Logistic regression test did not show a significant difference between the two groups in terms of the frequency of fluconazole susceptible strains in comparison to resistance and dose-dependent susceptible strains (*P*=0.181).

The results of Fisher's exact test showed a significant difference between the two groups in terms of itching, discharge, and pH ≥ 4.5. Accordingly, the improvement of vaginal symptoms and pH in the probiotic group was more considerable than that in the control group (Table 3). Finally, the adjusted logistic regression model indicated that the infection rate (positive culture) in the control group was higher (odd ratio=2.99) but not significant (*P*=0.059). When patients with resistance and dose-dependent strains were excluded, positive culture in the control group was higher (OR=4.7) than the probiotic group with a significant difference (*P*=0.025) (Table 4).

**Table 3:** Intergroup comparison of complaints and vaginal PH improvement

Symptoms	Fluconazole and probiotic (n=38)			Fluconazole and placebo (n=32)			P-value*
	Improved NO (%)	Unchanged NO (%)	Worsened NO (%)	Improved NO (%)	Unchanged NO (%)	Worsened NO (%)	
Vulvar burning	23 (60.5)	15 (39.5)	0 (0)	17 (53.1)	14 (43.8)	1 (3.1)	0.626
Vaginal discharge	29 (76.3)	9 (23.7)	0 (0)	13 (40.6)	18 (56.2)	1 (3.1)	0.004
Vulvar itching	29 (76.3)	9 (23.7)	0 (0)	15 (46.9)	16 (50)	1 (3.1)	0.017
Dyspareunia	14 (36.8)	24 (63.2)	0 (0)	15 (46.9)	16 (50)	1 (3.1)	0.390
PH<4.5	13 (34.2)	23 (60.5)	2 (5.3)	10 (32.3)	12 (38.7)	9 (29)	0.022

* fisher's exact test

**Table 4:** Logistic regression analysis of negative culture (mycological response) in patients with vulvovaginal candidiasis (age, relapse history, sexual activity, and contraceptive method were modulated)

Outcome	Crude OR (95% CI)	*P-value*	Adjusted OR	*P-value*
Negative culture	1.923	0.184	2.994	0.059
Negative culture*	2.281	0.129	4.755	0.025

*Patients with dose-dependent susceptible and resistant isolates were excluded.

## Discussion

Results of this study clarified that supplementary treatment with probiotics led to a significant improvement in clinical symptoms of major VVC, such as burning, discharge, and itching. Moreover, mycological cure that was considered negative culture in the probiotic supplementation group was more prevalent in the probiotics group, compared to the control group although the difference between these two groups was insignificant (*P*=0.184). 

In total, 16 patients (41%) with fewer than four episodes during the previous year, who were treated with a single dose of 150 mg fluconazole, had positive culture (44% vs. 56%, *P*=0.291) in probiotic and placebo groups. However, in a study performed by Martinez et al., the rate of positive culture in patients treated with a single dose of fluconazole was 10.3%, while that of the probiotic and placebo group was 38.5% [ 9 ].

The rate of mycological cure for all patients was 58.5%, which was close to the results of a study performed by Sobel et al. (1995) that reported 63% mycological cure after 35 days of treatment with an oral single dose of fluconazole. However, based on their findings, the corresponding rates for the patients with four or more episodes during the past year were 43% and %69 for fewer episodes [ 37 ]. Nevertheless, in the present study, positive culture was equal in patients with fewer and more than four episodes during the previous year. This consistency could be justified by the fact that the patients with four or more episodes were considered cases of recurrent VVC; therefore, they were treated with three doses of fluconazole every 72 h.

In a study performed in Iran, Sekhavat et al. reported a remarkable mycological and clinical response (98.6%) to an oral single dose of fluconazole in women with acute VVC [ 38 ]. Fluconazole is one of the most commonly prescribed drugs for *Candida* infections but some *Candida* species, such as *C. glabrata*, exhibited a low intrinsic susceptibility to azole derivatives. Moreover, several studies demonstrated the ability of *Candida* species for developing resistance during exposure to azoles [ 39 , 40 ]. More importantly, each isolate has a unique susceptibility pattern based on the infection site, and this issue was confirmed by several studies that indicated *Candida* isolates from oropharyngeal candidiasis were more resistant than candidemia isolates [ 41 , 42 ]. Badiee et al. (2011) performed a study in the south of Iran and found that *C. glabrata* strains were more resistant than *C. albicans* strains (45.5% vs. 10.5%) [ 43 ].

According to CLSI document M27-A3 guidelines, *Candida* species with MICs ≥ 64 μg/ml and MICs 16-32 μg/ml are defined as *Candida* resistant and dose-dependent, respectively, which could play the role of a confounding factor, while these issues have been ignored in more studies. Therefore, the elimination of possible limitations was conducted by *Candida* identification and antifungal susceptibility to fluconazole. Moreover, for better colonization, probiotics were administrated both orally and intravaginally, and only patients with positive culture were included in the present study. 

Soble et al. (2003) in their study demonstrated that clinical and mycological responses were more prevalent in patients with complicated vaginal candidiasis due to isolates with MICs ≤ 1 µg/ml in the baseline, compared to those of isolates with MICs&gt;1µg/ml at 35-day follow-up. Despite higher rates of clinical and mycological cures in patients with MICs ≤ 1µ g/ml, this difference was not statistically significant [ 44 ]. In the present study, MIC50 (1 µg/ml) was similar among the two groups. However, fluconazole resistance and dose-dependent isolates in the placebo group were more than the intervention group (21.1% vs. %9.4) but there was no significant difference between the two groups in this regard.

The results of this study indicated that clinical response to mycological response significantly improved in both groups. This was not unexpected since both groups underwent fluconazole therapy which is defined as a choice drug with the least toxicity and optimal compliance in patients who have experienced more than one episode of VVC [ 45 , 46 ].

Vaginal discharge and burning were the most common symptoms in both groups, which is similar to the results of other studies [ 13 , 40 ]. In a previous study conducted in Kashan, itching was reported as the most common symptom and vaginal discharge was only reported in 34% of patients [ 33 ]. Major studies about probiotic supplement therapy have generally focused on bacterial vaginitis and regarded probiotics as a supportive treatment. Therefore, only a few studies have investigated the role of probiotics in the treatment of VVC. Findings of some of these studies have indicated that probiotics are effective for the treatment of vaginal candidiasis [ 9 , 22 , 25 , 29 , 47 , 48 ].

In other studies, the role of probiotics, as a complementary treatment, is not promising and remains a controversial issue [ 13 , 23 , 32 ]. Moreover, the placebo-controlled trial studies about the assessment of fluconazole plus probiotics in vaginal candidiasis are limited [ 9 , 48 ]. In a randomized double-blind clinical trial, Martinez et al. compared the effectiveness of the administration of a single dose of fluconazole plus probiotic (Lactobacillus rhamnosus GR-1 and Lactobacillus reuteri RC-14) and fluconazole plus placebo for the treatment of vaginal candidiasis. According to their findings, concomitant use of probiotics with fluconazole played a significant role in the reduction of vaginal discharge associated with other symptoms, such as itching, burning, and dyspareunia, compared to the control group (10.3% vs. 34.6%; *P*=0.03) and positive culture (10.3% vs. 38.5%; *P*= 0.014) [ 9 ].

The current study is similar to the one carried out by Martinez et al. as both groups were treated with fluconazole [ 9 ]. After identification of *Candida* species, they found that the prevalence rates of non-albicans species in the probiotic and placebo group were 6.7% and 15.4% respectively. While in the present study, these rates were 21.1% and 21.9% for the probiotic and placebo groups, respectively [ 9 ]. However, neither of these studies were influenced by non-albicans species nor had a similar finding in terms of the significant improvement of vaginal symptoms in the probiotic group, compared to the placebo group. The similarity between the two studies, in terms of fluconazole therapy, study populations, and duration of the intervention could be one of the main reasons for the similarity with the study conducted by Martinez et al. [ 9 ].

Nouraei et al. [ 48 ] in a double-blind clinical trial study showed that vaginal candidiasis treatment with fluconazole plus oral probiotics supplementation in comparison to the probiotic supplementation group had an equal effect on the reduction of complaints and symptoms. However, the probiotic supplementation had better therapeutic effects on elimination and recovery time in vaginal candidiasis. Afrakhteh et al, in a double-blind clinical trial study, demonstrated that vaginal candidiasis treatment with clotrimazole and clotrimazole plus probiotics had equal outcomes and positive responses in the two groups (52.5% and 56.3%, respectively) (*P*=0.499). Moreover, in the aforementioned study, among different signs, only improvement of inflammation and redness had a significant difference [ 13 ].

In another study, Wagner et al. declared that infections due to *C. albicans* are correlated with the induction of pro-inflammatory responses in vaginal epithelial cells. However, according to them, estrogen and lactobacilli suppress the expression of NF-κB-related inflammatory genes and modulate the morbidity of *C. albicans* through modification of cytokine production by vaginal epithelial cells [ 49 ]. In their study, Murina et al. noted that fluconazole plus probiotics have a synergistic effect through maintenance of homeostasis and balance in vaginal and intestinal flora [ 50 ].

In the current study, the mycological cure was improving among the probiotic supplementation group but this improvement was insignificant. However, when patients with susceptible dose-dependent and resistant strains were excluded, the rate of mycological cure in the probiotics group was higher and had a significant difference while this form of assessment had been neglected in other studies. This exclusion could be one of the major reasons why probiotic supplementation therapy had different outcomes whereas complementary therapy for VVC became controversial in more studies.

## Conclusion

The results of the current study indicated that the administration of probiotics as a complementary treatment with fluconazole was effective. More specifically, it plays a principal role in the elimination of signs and symptoms of VVC as well as the assessment of antifungal susceptibility of *Candida* species isolated from vaginal candidiasis besides treatment with probiotics. This could lead to a bright future on the horizon of a complementary treatment with probiotics.

## Acknowledgments

The authors would like to thank all the patients who participated in this research. This article was derived from a thesis submitted in partial fulfillment of the requirement for the G.P degree. It should be mentioned that this study was supported by a research fund from Kashan University of Medical Sciences (Grant #95030). The authors are also grateful to Zist-Takhmir Pharmaceutical Company for their valuable advice and preparation of probiotics and placebo capsules.

## Authors’ contribution

M.N. designed and managed the study. Z.V., P.T., Z.D., R.CH., and T.F. performed the specimen collection. M.A. performed the antifungal susceptibility tests. M.S. contributed to statistical analysis. M.N. and I.H. prepared the manuscript.

## Conflicts of interest 

The authors declare that there was no conflict of interest in this study.

## Financial disclosure

This study was supported by a research fund from Kashan University of Medical Sciences (Grant #95030).
